# The RNA Domain Vc1 Regulates Downstream Gene Expression in Response to Cyclic Diguanylate in *Vibrio cholerae*

**DOI:** 10.1371/journal.pone.0148478

**Published:** 2016-02-05

**Authors:** Ankunda T. Kariisa, Kevin Weeks, Rita Tamayo

**Affiliations:** 1 Department of Microbiology and Immunology, University of North Carolina Chapel Hill, Chapel Hill, North Carolina, United States of America; 2 Department of Chemistry, University of North Carolina Chapel Hill, Chapel Hill, North Carolina, United States of America; East Carolina University School of Medicine, UNITED STATES

## Abstract

In many bacterial species, including the aquatic bacterium and human pathogen *Vibrio cholerae*, the second messenger cyclic diguanylate (c-di-GMP) modulates processes such as biofilm formation, motility, and virulence factor production. By interacting with various effectors, c-di-GMP regulates gene expression or protein function. One type of c-di-GMP receptor is the class I riboswitch, representatives of which have been shown to bind c-di-GMP *in vitro*. Herein, we examined the *in vitro* and *in vivo* function of the putative class I riboswitch in *Vibrio cholerae*, Vc1, which lies upstream of the gene encoding GbpA, a colonization factor that contributes to attachment of *V*. *cholerae* to environmental and host surfaces containing N-acetylglucosamine moieties. We provide evidence that Vc1 RNA interacts directly with c-di-GMP *in vitro*, and that nucleotides conserved among this class of riboswitch are important for binding. Yet the mutation of these conserved residues individually in the *V*. *cholerae* chromosome inconsistently affects the expression of *gbpA* and production of the GbpA protein. By isolating the regulatory function of Vc1, we show that the Vc1 element positively regulates downstream gene expression in response to c-di-GMP. Together these data suggest that the Vc1 element responds to c-di-GMP *in vivo*. Positive regulation of *gbpA* expression by c-di-GMP via Vc1 may influence the ability of *V*. *cholerae* to associate with chitin in the aquatic environment and the host intestinal environment.

## Introduction

Cyclic diguanylate (c-di-GMP) is a ubiquitous second messenger important for bacterial adaptation to environmental conditions. In response to largely undefined signals at the cell surface, changes in intracellular c-di-GMP concentration relays that information to target intracellular effectors, regulating processes such as biofilm formation, motility and virulence gene expression (reviewed in [1]). The intracellular level of c-di-GMP is controlled by the activities of diguanylate cyclases (DGCs) and phosphodiesterases (PDEs), enzymes responsible for the synthesis and degradation of c-di-GMP, respectively [2–8]. Bacterial genomes often contain multiple genes encoding putative DGC and PDE enzymes, potentially reflecting a requirement for tight regulation of c-di-GMP levels and the need to modulate c-di-GMP in response to diverse extracellular cues.

Various classes of intracellular c-di-GMP receptors have been identified and can control c-di-GMP regulated processes via transcriptional, post-transcriptional or post-translational mechanisms. In addition to numerous protein sensors of c-di-GMP, post-transcriptional regulation by c-di-GMP can occur through riboswitches, *cis* acting regulatory elements found in the 5’ untranslated region (UTR) of some mRNAs [9–11]. Two types of c-di-GMP sensing riboswitches have been identified, class I and class II [9,12]. The two classes share no structure or sequence homology. The class I c-di-GMP riboswitches contain a GEMM motif, which is widespread in bacteria and so named because it often resides in the 5’ UTR of genes with functions predicted to relate to the environment, membrane or motility [9,13]. Based on the co-crystal structure of c-di-GMP with the *V*. *cholerae* aptamer Vc2, GEMM motifs are predicted to contain two adjacent stem-loops, termed P2 and P3, a tetraloop-tetraloop receptor motif that stabilizes the interaction between P2 and P3, and a P1 stem that forms through base pairing between the flanking 5’ nucleotides of P2 and the flanking 3’ nucleotides of P3 [9,11,13]. The nucleotides of Vc2 that contact c-di-GMP lie at the junction of P1, P2, and P3.

Only a few studies have directly addressed the functionality of a c-di-GMP riboswitch *in vivo*, in its native genetic context, and the impact of c-di-GMP sensing on the physiology of a bacterium. *Clostridium difficile* encodes numerous putative c-di-GMP riboswitches (both class I and class II), some of which lie upstream of genes encoding surface proteins and organelles such as flagella and Type IV pili [9,12]. Artificial elevation of c-di-GMP in *C*. *difficile* represses flagellar gene expression and swimming motility [14–16]. These findings are consistent with prior work showing that the GEMM riboswitch Cd1 upstream of a flagellar operon functions as an “off switch” in response to c-di-GMP; mutations in Cd1 that impair its interaction with c-di-GMP result in increased reporter gene expression in a heterologous bacterial host [9]. Conversely, the class II riboswitch upstream of a pilin gene functions as an “on switch” in response to c-di-GMP in *C*. *difficile* [17]. C-di-GMP positively regulates type IV pilin gene expression through direct interaction with the riboswitch, promoting cell aggregation, biofilm formation and surface motility [17,18]. Recently, the production of a zinc metalloprotease, ZmpI, and one of its targets, the *C*. *difficile* surface protein CD2831, have been shown to be regulated by c-di-GMP *in vivo* [19]; these findings are consistent with the presence of class II c-di-GMP riboswitches upstream of the respective genes [12,20].

The biochemistry and function of the second predicted GEMM riboswitch in *V*. *cholerae*, called Vc1, has not been examined. The predicted GEMM motif of Vc1 has 75% overall identity with the well-characterized Vc2 GEMM motif, with 85% identity in the region that encodes the first stem-loop, P2. The residues that contact the c-di-GMP ligand in Vc2 (G20, A47 and C92) are conserved in Vc1 (G12, A39, and C104, respectively). The nucleotides that form the interhelical Watson-Crick base pair, universally conserved among GEMM riboswitches, are also present in Vc1 (C36 and G90 in P2 and P3 of Vc1, respectively). While critical features of the GEMM riboswitch are conserved in Vc1, the differences between Vc2 and Vc1 may help define broadly the mechanisms by which c-di-GMP acts through GEMM riboswitches.

Vc1 lies upstream of *gbpA*, which encodes a well-characterized colonization factor that is well conserved among *V*. *cholerae* environmental and clinical isolates [21]. GbpA is a secreted protein that aids in colonization of surfaces in aquatic environments (the natural habitat of *V*. *cholerae*) and the small intestine (the tissue colonized by pathogenic *V*. *cholerae*) [22]. GbpA recognizes chitin, a polymer of N-acetylglucosamine (GlcNAc) found in the exoskeletons of zooplankton and crustaceans colonized by *V*. *cholerae* in aquatic reservoirs [22]. GbpA also plays a role in colonization of the small intestine by interacting with GlcNAc present in mucin and on the surface of intestinal epithelial cells [22,23]. *In vitro* studies have shown that GbpA selectively interacts with GlcNAc oligomers and polymers [24]. Accordingly, *V*. *cholerae* mutants lacking *gbpA* are attenuated both in an animal model of infection and attachment to chitinous surfaces [22,23]. Studying Vc1 may thus provide insight about the role of c-di-GMP and Vc1 in modulating attachment of *V*. *cholerae* to environmental and host surfaces.

In this study, we combine biochemical and genetic approaches to evaluate the Vc1 element for function as a riboswitch that controls *gbpA* expression in response to c-di-GMP. We provide evidence that Vc1 directly interacts with c-di-GMP *in vitro*, and interfering with Vc1 sensing of c-di-GMP impairs downstream gene expression *in vivo*. Furthermore, using a Vc1 reporter gene fusion under the control of a heterologous, constitutive promoter shows that Vc1 is a c-di-GMP responsive RNA element. These data suggest that c-di-GMP signaling through Vc1 promotes *gbpA* expression and GbpA-dependent adherence to host and environmental surfaces.

## Materials and Methods

### Growth conditions and media

*V*. *cholerae* C6706 and isogenic mutant strains (S1 Table) were cultured at 37°C in Luria-Bertani (LB) broth containing 100 μg/ml streptomycin (Sm), 10μg/ml chloramphenicol (Cm), and/or 50 μg/ml ampicillin (Amp), as appropriate.

### Artificial manipulation of intracellular c-di-GMP

The manipulation of the intracellular level of c-di-GMP in *V*. *cholerae* was achieved as described previously [25,26]. Briefly, overnight cultures of *V*. *cholerae* harboring pBAD33 (“vector”), pBAD33::*vieA* (“pPDE”), pBAD33::*vieA-E170A* (“pPDE^mut^”) or pBAD33::VCA0956 (“pDGC”) were back-diluted 1:100 in LB-Cm broth and grown at 37°C with shaking. Induced cultures contained 0.2% L-arabinose unless otherwise specified. Samples were collected for western blotting, β-galactosidase assays and/or qRT-PCR analysis as described below.

### 5’ Rapid Amplification of cDNA Ends (RACE)

RNA was collected using TRIsure (Bioline) and the RNeasy kit (Qiagen) as described previously [27]. A *gbpA* specific primer, gbpAsp1, was used to make cDNA from RNA in a reverse transcription reaction with the Tetro cDNA Synthesis Kit (Bioline), using the manufacturer’s protocol. Next, Terminal transferase, TdT (NEB), was added to the reaction to introduce a homopolymeric A sequence to the 5’ end the cDNA molecules, using the manufacturer’s protocol. PCR using nested primers specific to the 5’ homopolymeric tail (Race1g, Race1c, Race1a) and *gbpA* (gbpAsp2) were used to amplify the cDNA products. Sequencing of amplified cDNA products identified the +1 site of transcription.

### Genetic manipulations

Strains and plasmids used in this study are listed in S1 Table. All oligonucleotide primers used for cloning are listed in S2 Table. Details regarding the generation of strains used in this study are described in the Supplemental Methods (S1 File).

### GbpA antibody production

Anti-GbpA antiserum was produced by Yenzym 192 Antibodies, LLC, South San Francisco, CA. Antiserum was raised in rabbits to a synthetic peptide (CSNATQYQPGTGSHWEMAWDKR) that corresponds to GbpA from *V*. *cholerae*. The animal facilities were NIH/OLAW/PHS assured, USDA certified, and IACUC regulated.

### Western blot analysis

Overnight cultures were diluted 1:100 and grown in LB broth at 37°C with aeration until mid-exponential phase (OD_600_ ~ 0.6–0.8). Equal volumes of supernatant, normalized to OD_600_, were collected, and proteins were precipitated using 10% trichloroacetic acid (TCA). TCA-precipitated samples were separated by electrophoresis, transferred onto nitrocellulose membranes, and probed with rabbit anti-GbpA antibodies. Goat anti-rabbit IgG conjugated with IR800 dye (Thermo Scientific) was used as the secondary antibody. Membranes were imaged using an Odyssey imaging system (LI-COR). At least three independent experiments were performed, and a representative image is presented. Densitometry analyses were carried out using Odyssey software by normalizing the intensities of the bands corresponding to GbpA to those of a cross reactive band that did not change intensity in any of the strains or conditions tested (indicated by asterisks in relevant images).

### *In vitro* transcription

For SHAPE analysis, *V*. *cholerae* C6706 genomic DNA was used as the template in PCR with primers T7linkF + T7R, yielding a product consisting of the T7 promoter and the -15 to +665 portion of the *gbpA* transcript. The PCR product was used as template for *in vitro* transcription of the RNA using the Ambion MEGAscript^®^ T7 Kit, according to the manufacturer’s instructions. The RNA was precipitated with ethanol and suspended in water. RNAs were resolved on a denaturing 6% polyacrylamide gel (1X TB, 7M urea, 6% acrylamide). The desired RNA bands were excised from the gel and placed in RNase-free water overnight at 4°C to elute the RNA. The eluted material was ethanol precipitated to recover RNA, and the RNA was suspended in TE buffer.

For equilibrium dialysis, the templates used for the transcription reactions were amplified from genomic DNA of C6706 and Vc1 mutant derivatives using T7Vc1F + Vc1R3. Vc2^WT^ and Vc2^G20T^ were amplified from C6706 genomic DNA and pCVD442::Vc2^G20T^, respectively, using T7Vc2F + T7Vc2R. RNA was transcribed from the resulting DNA as described above, yielding products with the T7 promoter and the -15 to +140 region of the *gbpA* 5’ UTR (Vc1 and Vc1^G12U^; numbering according to 5’ RACE results) or the -2 to +209 region of the VC1722 transcript (Vc2 and Vc2^G20U^; numbering according to the 3IRW entry in PDB).

### SHAPE analysis of the *gbpA* mRNA

Vc1 RNA (10 μl, 7 μM) was denatured by heating at 95°C for 2 minutes and snap-cooled on ice for 2 minutes. RNA (2 μl) was combined with 6 μl of 3.3× folding buffer (33 mM HEPES, 333 mM MgCl_2_, 333 mM NaCl, pH 8), 2 μl of water and either 10 μl of water or 10 μl of 1 mM c-di-GMP [28]. The RNA was folded at 37°C for 30 min. Folded RNA (9 μl) was added to 1 μl of 30 mM 1M7 (in DMSO) or to 1 μl DMSO only, immediately mixed thoroughly, and incubated at 37°C for 3 min. RNA was precipitated with ethanol and suspended in TE buffer. SHAPE adducts were detected by primer extension as described [29]. 1M7 and DMSO treated RNA, as well as untreated RNA (later used for sequencing), were mixed with fluorescently labeled DNA oligonucleotides that anneal to *gbpA* (Vc1SHAPE_R). Primer extension was initiated by addition of Superscript III reverse transcriptase (Invitrogen). Sequence information was generated using unmodified RNA by performing primer extension in the presence of dideoxynucleotide triphosphates (ddNTPs). Primer extension products were ethanol precipitated and suspended in formamide. Products were resolved on an ABI 3500 capillary electrophoresis instrument and analyzed using QuShape [30]. Each experiment was performed at least three times, and statistical significance was determined using Student’s t-test.

### Synthesis of radiolabeled c-di-GMP

Radiolabeled c-di-GMP was generated as detailed previously [7,14]. Briefly, c-di-GMP^32^ was synthesized in vitro using recombinant diguanylate cyclase WspR (from *Pseudomonas aeruginosa*) [31], with [α-^32^P]GTP as the substrate (Perkin-Elmer Life Sciences). The presence and yield of the c-di-GMP^32^ reaction product was confirmed by thin layer chromatography, and c-di-GMP^32^ was purified using Ultrafree-MC 5000 Da molecular weight cut-off columns (Centricon) [7].

### Equilibrium dialysis

*In vitro* transcribed Vc1, Vc1^G12U^, Vc1^A39U^, Vc1^C104G^, Vc2 and Vc2^G20U^ RNA were denatured by heating at 95°C for 2 minutes and snap-cooled on ice for 2 minutes. RNA was then combined with 4 μl of 5X folding buffer (50 mM HEPES, 500 mM MgCl_2_, 500 mM NaCl, pH 8) and added in a 20 μl total volume to chamber A of a two-chamber equilibrium dialysis device with a 5000 Da MWCO membrane (Dispo Equilibrium Dialyzer, Harvard Apparatus). c-di-GMP^32^ was combined with 4 μl of 5× folding buffer and added in a 20 μl total volume to chamber B of the equilibrium dialysis device. The final concentrations of RNA and ligand in the binding reactions were: 3.33 nM c-di-GMP^32^, 30 μM Vc1 and mutant derivatives, and 10 μM Vc2 and Vc2^G20U^. Samples were allowed to equilibrate for 22 hours, and then 2 μl were removed for scintillation counting. For each RNA, the products of two separate transcription reactions were tested in triplicate, and the data were combined. For competition experiments, following 10 hours of equilibration with ligand (or controls), the contents of chamber B were removed and replaced with 100 μM GTP or 100 μM c-di-GMP diluted in 5X folding buffer. Samples were allowed to equilibrate for an additional 10 hours, then 2 μl were removed for scintillation counting. Binding was calculated as the percentage of the total radioactivity present in the chamber (A) containing RNA. If no binding of c-di-GMP^32^ occurs, c-di-GMP^32^ will distribute equally between the two chambers; if RNA capable of binding c-di-GMP^32^ is present, c-di-GMP^32^ will accumulate in that chamber. Each experiment was repeated independently at least three times.

### RNA isolation and quantitative real-time PCR

Transcriptional analyses by quantitative reverse transcriptase PCR (qRT-PCR) were done essentially as previously described, using mid-exponential phase (OD_600_ ~ 0.5–0.7) cultures [27]. For cDNA synthesis, 200 ng of RNA was reverse transcribed using the Tetro cDNA Synthesis Kit with random hexamers as primers (Bioline). As negative controls, reactions were run without reverse transcriptase. cDNA samples were combined with 2× SYBR/fluorescein mix (SensiMix; Bioline), 7.5 μM of each primer (RPB2F + RPB2R for *rpoB*, gbpAqF2 + gbpAqR2 for the *gbpA* ORF) (S2 Table). The reactions were run using a MyiQ thermocycler (Bio-Rad) and the following program: 95°C for 10 min, followed by 40 cycles of 95°C for 30 s, 55°C for 1 min, and 72°C for 30 s. Melt curves were performed to verify the amplification of single products, and all values were adjusted for primer binding efficiency, as calculated from a standard curve of C6706 genomic DNA. Data were analyzed using the ΔΔCt method, with values normalized to the specified reference strain/condition, and transcript levels normalized to those of the housekeeping gene *rpoB* in each sample. For each strain a minimum of three biological samples was tested. Statistical significance was determined by unpaired t-test.

### Beta-galactosidase assays

Dilutions of stationary phase cultures (grown 12–16 hours at 37°C with aeration) were assayed for β-galactosidase activity using ortho-nitrophenyl-β-D-galactoside as a substrate as described previously [32]. At least three independent experiments were done and the data were combined and analyzed by unpaired t-tests.

## Results

### The predicted structure and c-di-GMP binding sites of Vc1

**Fig 1:**
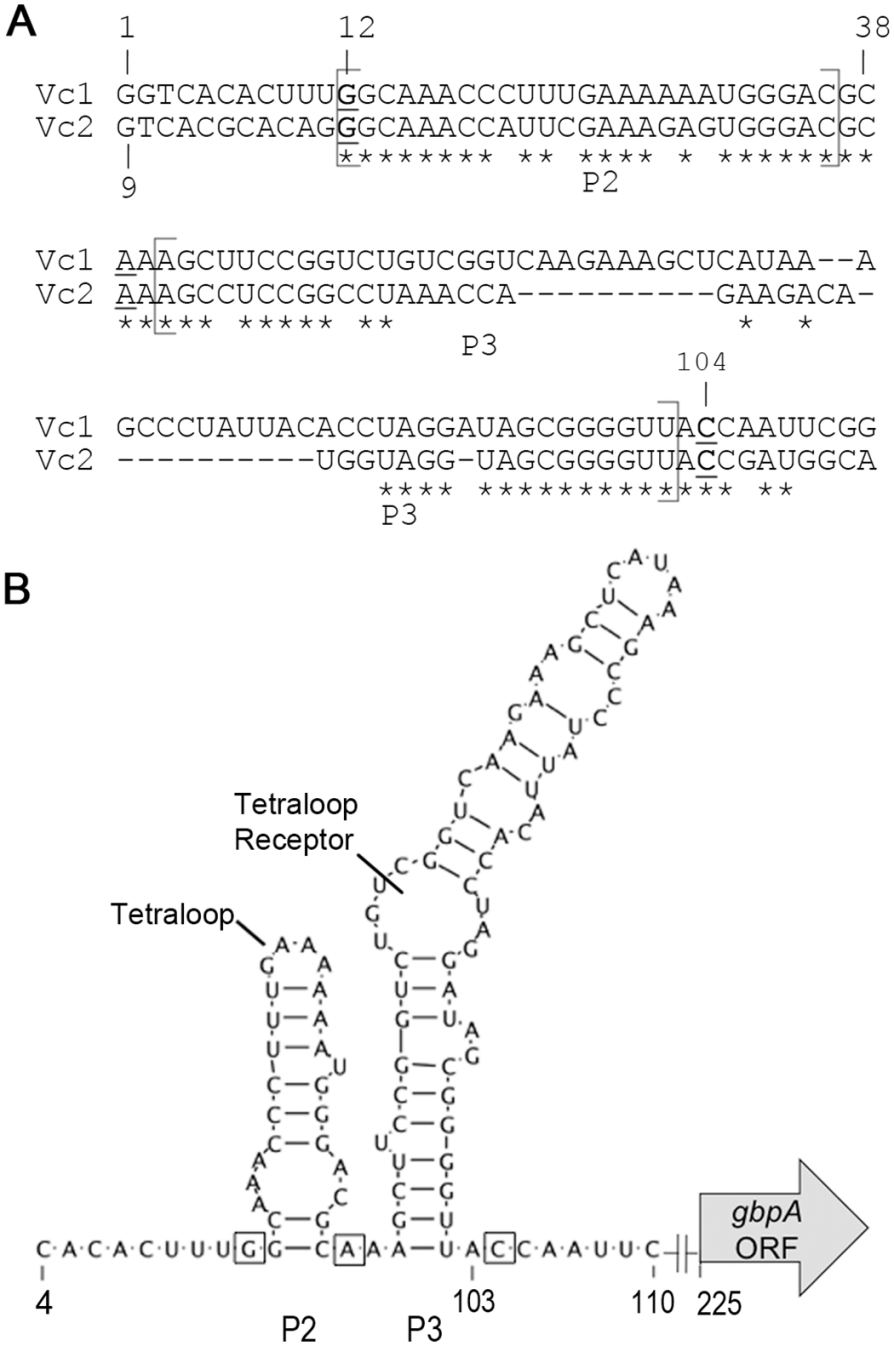
Vc1 secondary structure and putative contact residues for c-di-GMP. (A) The +1 site of transcription, which is 244 base pairs upstream of the experimentally determined translational start site (S1 Fig), was identified using 5’ RACE. Vc2 and Vc1 were aligned using ClustalW2. The alignment begins with the +1 transcriptional start site for Vc1 and the nucleotide at position +9 according to the sequence annotation for Vc2 (PDB 3IRW [11]). Asterisks represent nucleotides that are conserved between Vc1 and Vc2. Nucleotides bolded and underlined are contact residues for c-di-GMP in Vc2 and are predicted contact residues for c-di-GMP in Vc1. The regions predicted to encode P2 and P3 are labeled. (B) The predicted structure of Vc1 based on an alignment with Vc2 and the consensus GEMM aptamer structure. The predicted tetraloop and tetraloop receptor are noted.

To begin analyzing the Vc1 element predicted upstream of the *gbpA* open reading frame (ORF), we first defined the 5’ UTR of *gbpA* by identifying the transcriptional start site using 5’ Rapid Amplification of cDNA Ends (5’ RACE). To rule out potential differences in the length of the *gbpA* 5’ UTR in response to c-di-GMP, 5’ RACE was performed with RNA collected from strains with both wild type and low c-di-GMP levels. The intracellular level of c-di-GMP in *V*. *cholerae* was reduced by ectopic expression of a c-di-GMP PDE as described previously [33]. Briefly, *V*. *cholerae* harboring a plasmid that allows inducible expression of the well-characterized c-di-GMP PDE, VieA, was used to test the effect of depleting intracellular c-di-GMP [7,25,33]. *V*. *cholerae* containing vector only, treated identically to *V*. *cholerae* with pPDE, serves as an unperturbed “wild-type” c-di-GMP control. 5’ RACE showed that the transcriptional start site (+1) remains unchanged between *V*. *cholerae* with native and low c-di-GMP levels and is 225 nucleotides upstream of the annotated translational start site of *gbpA* (Fig 1A). The predicted GEMM motif of Vc1 is thus encoded within the first 111 nucleotides of the *gbpA* 5’ UTR. Using this information and the GEMM consensus sequence and structure, the RNA structure of the first 120 bases of the *gbpA* 5’UTR, which encompass the GEMM motif of Vc1, was modeled and is consistent with the accepted secondary structure of Vc2 (Fig 1A and 1B) [9,11].

### Mutations in predicted c-di-GMP contact residues of Vc1 affect downstream gene expression

**Fig 2:**
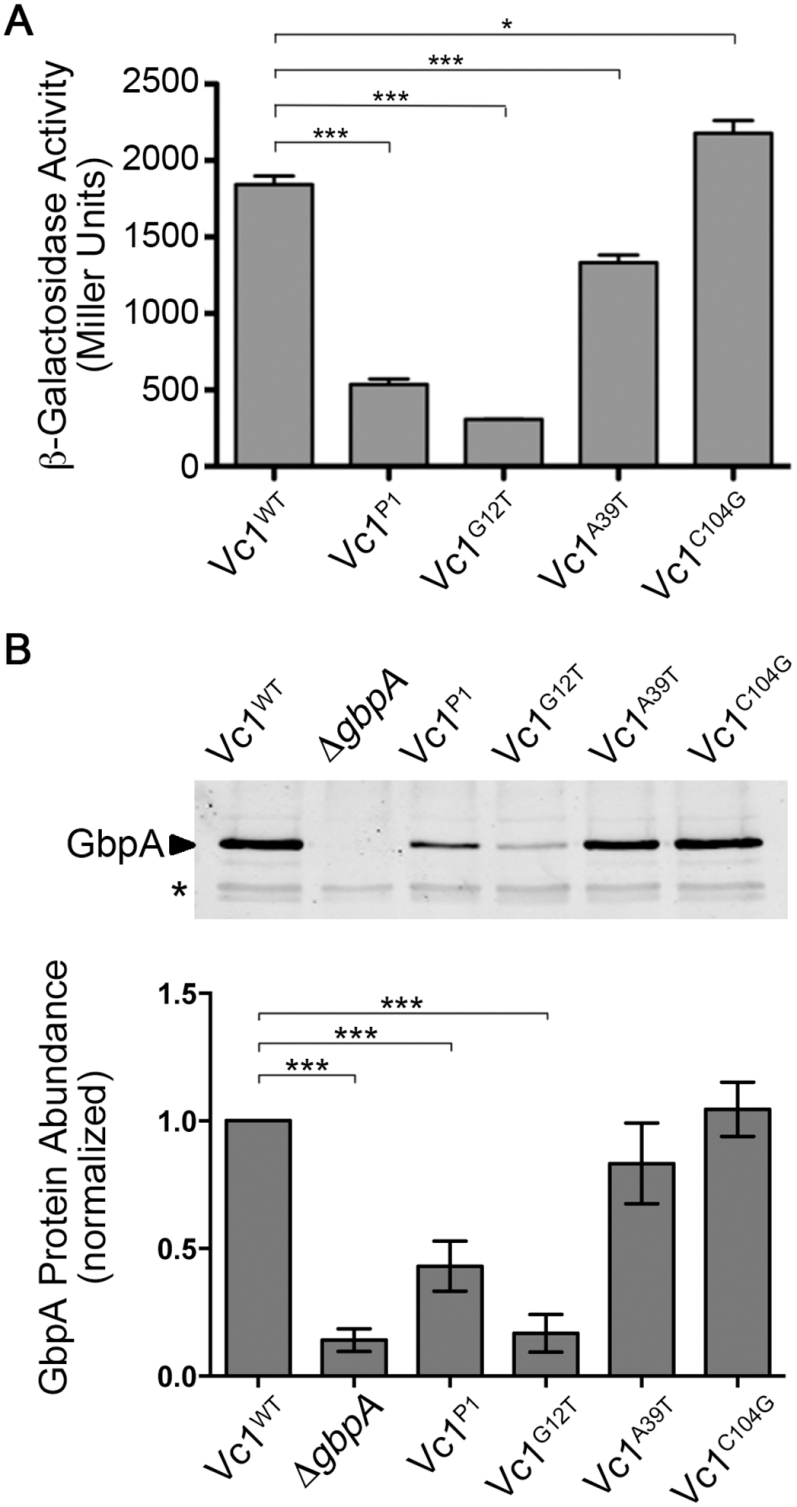
Vc1 influences downstream gene expression. (A) β-galactosidase activity of *V*. *cholerae* strains with plasmid-borne *lacZ* translational fusions to wild type Vc1 or mutant derivatives. Transcription initiation is controlled by the *lac* promoter. Three independent experiments were done, and the means and standard deviations are shown. *** *P* < 0.001, * *P* < 0.05 by unpaired t-test. (B) Western blot analysis of the GbpA levels in the supernatants of cultures of wild type *V*. *cholerae*, an isogenic Δ*gbpA* strain, or strains with mutations in Vc1 (P1, G12T, A39T, or C104G). The image shown is representative of three independent experiments. (C) Densitometry of the western blots was done by comparing the intensity of the GbpA band to that of a cross-reactive band in the same lane (indicated by an asterisk), and then normalizing to the wild type values. Shown are the means and standard deviations for three independent experiments. *** *P* < 0.001 by unpaired t-test.

To examine the role of Vc1 in regulating gene expression in response to c-di-GMP, we constructed a translational reporter consisting of the *gbpA* 5’ UTR fused to *lacZ* from *Escherichia coli* (S1 Fig). The heterologous promoter P_*lac*_ drove expression, allowing constitutive, P_*gbpA*_-independent transcription initiation during growth in rich medium. We generated four derivatives of the reporter plasmid, each with a mutation in Vc1 predicted to interfere with sensing c-di-GMP. The “Vc1^P1^” derivative contains mutations in five consecutive nucleotides in Vc1: C4G, A5T, C6G, A7T, C8G. In Vc2, these five nucleotides comprise the P1 stem that forms in the presence of c-di-GMP. The other three mutant reporters each contain a single point mutation in a nucleotide predicted to interact directly with the c-di-GMP ligand: G12T, A39T and C104G (noted in Fig 1A and 1B) [11]. The plasmids were introduced into *V*. *cholerae*, and reporter activity was measured using a β-galactosidase assay. Relative to the wild type reporter, the Vc1^P1^, G12T and A39T mutations significantly reduced, but did not eliminate, β-galactosidase activity (Fig 2A). These results suggest that G12 and A39 may be important for sensing c-di-GMP and for full expression of the downstream gene. Conversely, the C104G mutation caused a modest increase in β-galactosidase activity, suggesting it is not required for sensing c-di-GMP. An alternate mutation, C104A, was made, but this mutation also did not have an effect on β-galactosidase activity (data not shown).

We next assessed the functionality of Vc1 in its native genetic context. The four mutations described above, P1, G12T, A39T and C104G, were individually introduced into the chromosomal Vc1 in *V*. *cholerae* C6706 via allelic exchange, and GbpA production was measured by western blot. A strain with an in-frame deletion of *gbpA* served as a control. Consistent with the β-galactosidase reporter assays, the level of GbpA decreased significantly in the P1 and G12T mutants (Fig 2B and 2C). GbpA protein abundance was not significantly altered in the A39T and C014G mutants. In addition, the effects of each chromosomal point mutation on GbpA production mirrored the effects of the mutations in the extrachromosomal transcriptional reporter. Thus, although each of the equivalent nucleotides plays critical roles in c-di-GMP in Vc2 binding *in vitro*, the G12 mutation in Vc1 has the largest impact on downstream gene expression *in vivo*.

### Evidence that Vc1 interacts directly with c-di-GMP *in vitro*

To test whether Vc1 interacts with c-di-GMP, we used several approaches. First, we used selective 2’-hydroxyl acylation analyzed by primer extension (SHAPE) chemistry to probe the potential interaction between Vc1 and c-di-GMP [34]. SHAPE uses acylating agents, such as 1-methyl-7-nitroisatoic anhydride (1M7), which selectively form adducts at the 2’-hydroxyl group of an RNA nucleotide. The ability of 1M7 to react with a nucleotide depends on the local flexibility of that nucleotide within an RNA structure, and flexibility correlates with the extent to which a nucleotide is constrained within a structure [35]. SHAPE has been used successfully to assess the interactions between several riboswitches and their target ligands, including the Vc2 aptamer and c-di-GMP [29,36]. A comparison of structures for a GEMM riboswitch in the presence and absence of c-di-GMP has not been reported to date, and we used SHAPE chemistry to assess the differences between *apo*-Vc1 and Vc1 with c-di-GMP.

**Fig 3:**
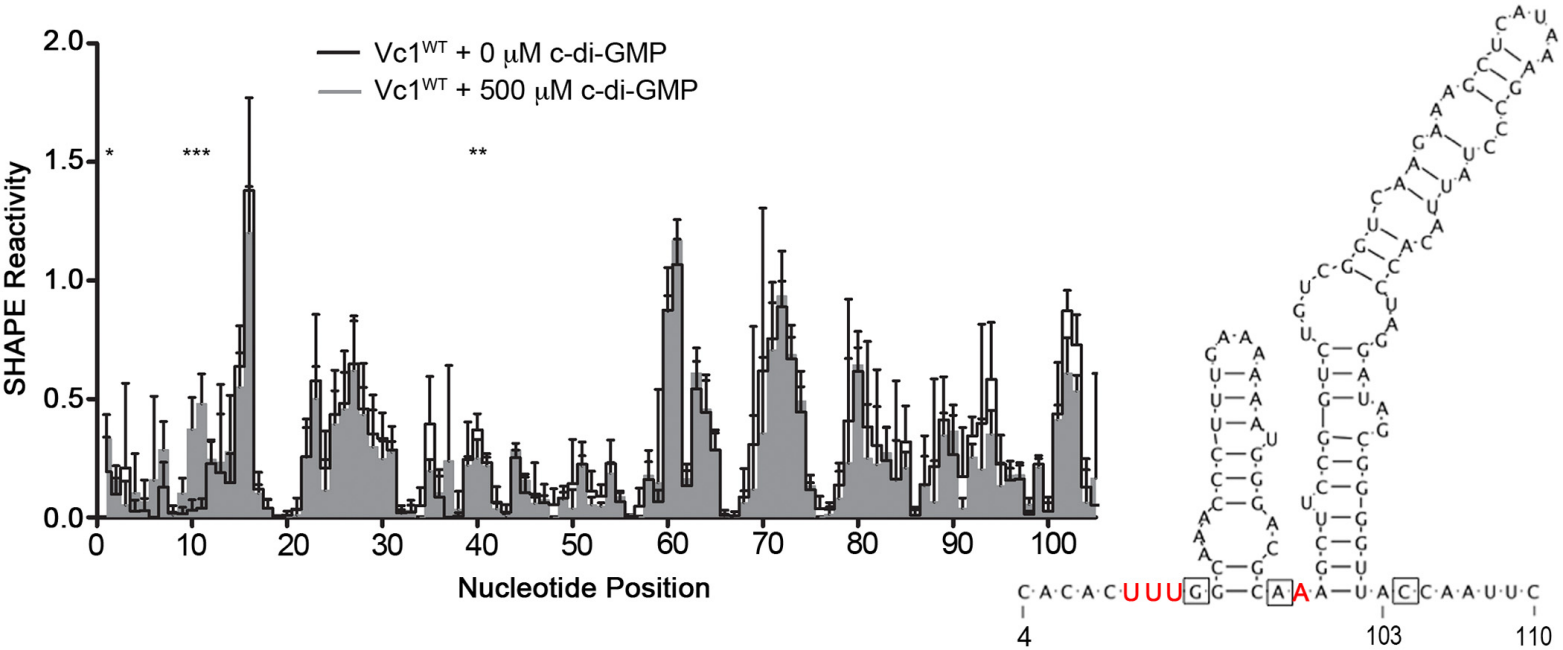
c-di-GMP impacts the SHAPE reactivity of specific regions in Vc1 RNA. Vc1 RNA transcripts were incubated with or without 500 μM c-di-GMP and analyzed by SHAPE. SHAPE reactivity values for each nucleotide position were obtained by averaging values from five independent experiments. The inset shows in red text the location of the nucleotides with significantly altered reactivity, mapped on the predicted structure of Vc1. The boxes denote the G12, A39 and C104 residues. * *P* < 0.05 by unpaired t-test comparing SHAPE reactivity values for each nucleotide position, with or without c-di-GMP, indicating significant changes in reactivity in response to c-di-GMP.

An *in vitro* transcript of the *gbpA* mRNA, encompassing the entire 5’ UTR of *gbpA* and 440 bases of the open reading frame (ORF), was folded in the presence or absence of c-di-GMP, subjected to 1M7 modification, and analyzed by SHAPE. An RNA sequence that extends downstream of the Vc1 aptamer was used because of the possibility that neighboring RNA sequence impacts the structure of the riboswitch. Statistically significant differences in reactivity were observed in three distinct regions of the GEMM motif in the presence and absence of c-di-GMP (Fig 3, asterisks). Reactivity of nucleotides U9, U10, and U11, located adjacent to the first predicted c-di-GMP contact residue G12, significantly increased in the presence of c-di-GMP. A40 showed a decrease in reactivity in the presence of c-di-GMP and is adjacent to the second predicted contact residue for c-di-GMP, A39. An increase in reactivity in the presence of c-di-GMP was also observed in region immediately 5’ of Vc1; in Vc2, this region contributes to the “P1” stem formed in the presence of c-di-GMP [9,11]. No changes were observed near or around C104. As a control, we performed SHAPE with Vc1 with and without another guanosine nucleotide, GTP. No statistically significant differences in Vc1 reactivity were observed in the presence of GTP relative to *apo*-Vc1, indicating that GTP and Vc1 do not interact (S2 Fig). Together these results suggest that c-di-GMP specifically interacts with Vc1 via the G12 and A39 regions, but not the C104 region, though indirect effects of c-di-GMP on the reactivity of the G12 and A39 regions are also possible.

**Fig 4:**
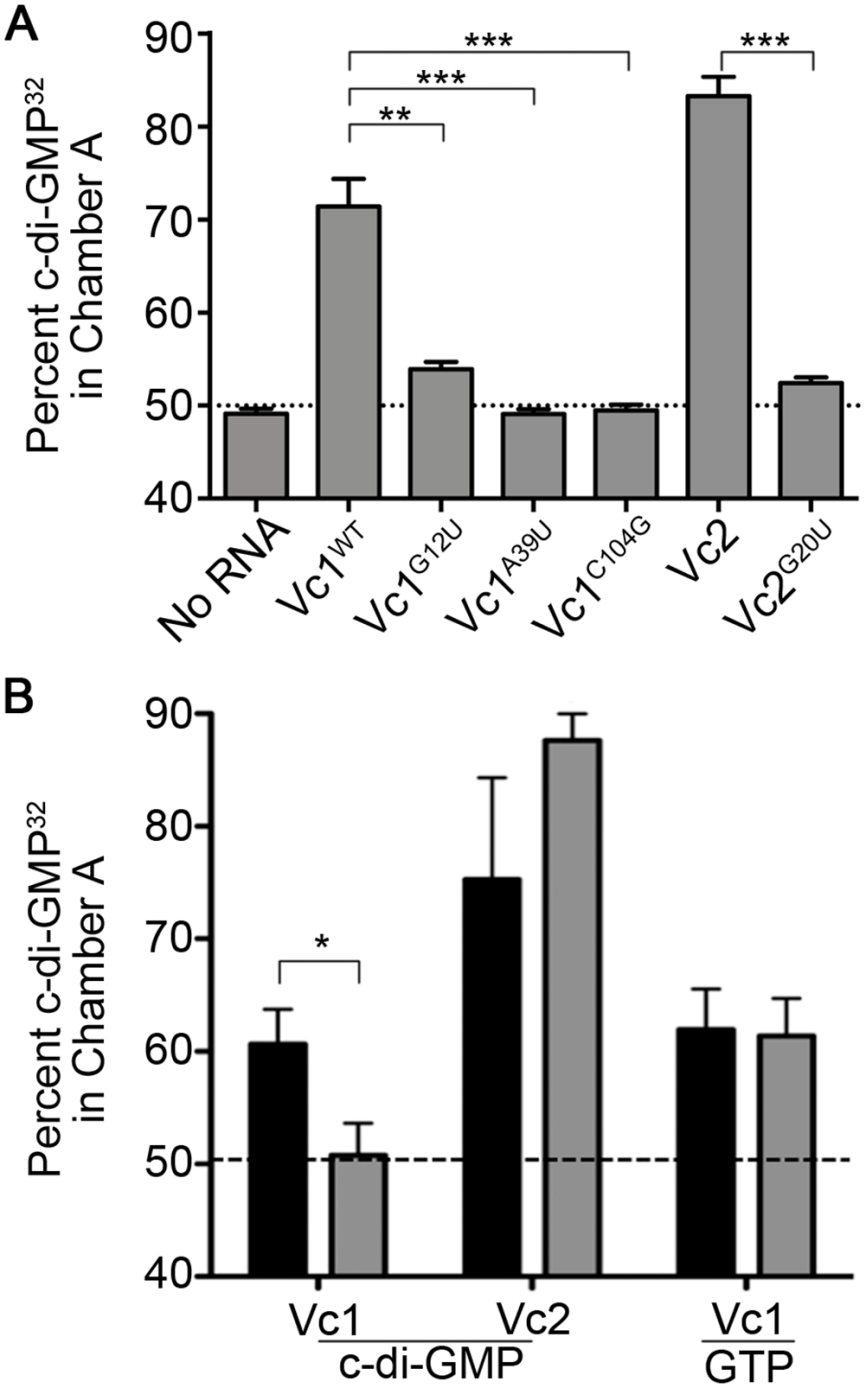
Vc1 directly and specifically interacts with c-di-GMP. (A) Binding of c-di-GMP^32^ to *in vitro* transcribed Vc1 RNA and mutant derivatives was measured by equilibrium dialysis as described in the Materials and Methods. Vc2 and Vc2^G20U^ RNA were included as controls, and no-RNA samples were used as a negative control. (B) The specificity of binding to c-di-GMP to Vc1 and Vc2 RNA was assessed by equilibrium dialysis, allowing c-di-GMP^32^ to bind and reach equilibrium as the first measurement (black bars), then competing with unlabeled, excess c-di-GMP or GTP and determining the percentage of c-di-GMP^32^ retained in the RNA chambers (grey bars). **P < 0.01, **P < 0.001 by unpaired t-test.

We next used equilibrium dialysis to assess the interaction between Vc1 RNA and c-di-GMP. As expected, when no RNA was added, c-di-GMP^32^ was detected equally in both chambers; Vc2 RNA, which served as a positive control, sequestered c-di-GMP^32^ (Fig 4A) [9–11,37]. Vc1 RNA also sequestered c-di-GMP^32^, but less so than Vc2 RNA (71% versus 83% of c-di-GMP in the RNA chamber, respectively), suggesting that Vc1 RNA binds c-di-GMP but perhaps with a lower affinity than Vc2 (Fig 4A).

We next evaluated the binding of c-di-GMP to the mutant Vc1 RNAs. Consistent with the *in vivo* data shown in Fig 2, the G12U mutation in Vc1 RNA significantly diminished sequestration of c-di-GMP, but did not fully eliminate it, unlike the equivalent mutation in Vc2 (Fig 4A). That the Vc1^G12U^ mutant RNA retained some ability to sequester c-di-GMP^32^ indicates that this residue is not absolutely required for c-di-GMP binding. However, the A39U and C104G mutations, which did not substantially impact GbpA production, abolished the ability of Vc1 to sequester c-di-GMP (Fig 4A).

We next examined the specificity of Vc1 for c-di-GMP using a two-step equilibrium dialysis binding experiment. Vc1, as well as the Vc2 positive control, were allowed to interact with radiolabeled c-di-GMP for 10 hours as described above, and the proportion of c-di-GMP sequestered in the RNA-containing chamber was measured. As above, Vc2 sequestered c-di-GMP^32^ to a greater extent than Vc1 (Fig 4B, black bars). For the second step, the non-sequestered radiolabeled c-di-GMP was removed, and excess non-radiolabeled (“cold”) c-di-GMP or GTP was added. After an additional 10 hours to allow binding and potential displacement of c-di-GMP^32^ from the first step, the remaining radioactivity associated with the RNA was assessed. For Vc1, the addition of unlabeled c-di-GMP, but not unlabeled GTP, competed with radiolabeled c-di-GMP for binding with Vc1 and shifted the distribution of c-di-GMP^32^, indicating that the interaction between c-di-GMP and Vc1 is specific (Fig 4B). Interestingly, addition of unlabeled c-di-GMP did not compete with c-di-GMP^32^ for Vc2 binding over the timescale of the dialysis assay, consistent with previous work showing that c-di-GMP dissociates very slowly from the Vc2 RNA [37]. Vc1 appears to have a faster off rate than Vc2, as c-di-GMP^32^ could be displaced by the added unlabeled c-di-GMP over the 10 hour time course of the assay.

### Decreasing intracellular c-di-GMP reduces Vc1-dependent gene expression

**Fig 5:**
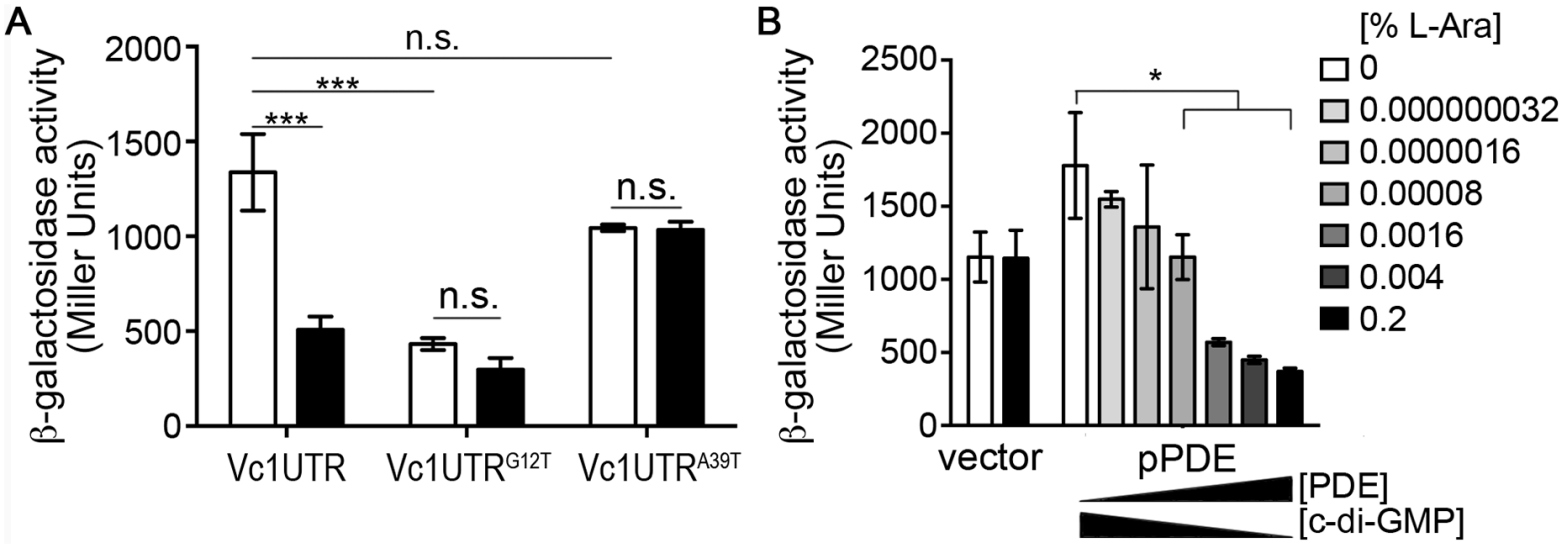
Lowering intracellular c-di-GMP reduces Vc1-dependent gene expression. β-galactosidase activity of *V*. *cholerae* strains with chromosomal, translational fusions of *E*. *coli lacZ* to the *gbpA* 5’ UTR with wild type Vc1, Vc1^G12T^, or Vc1^A39T^, with transcription initiation under the control of the constitutive P_*lacUV5*_ promoter: PlacUV5-Vc1UTR-*lacZ*, PlacUV5-Vc1UTR^G12T^-*lacZ*, PlacUV5-Vc1UTR^A39T^-*lacZ*, respectively [38]. Each reporter strain carried pPDE and was grown without arabinose (wild-type c-di-GMP level; white bars) or with 0.2% arabinose (reduced c-di-GMP; black bars). (B) Dose response analysis using the P_*lacUV5*_-Vc1UTR-*lacZ* reporter strain, with vector or pPDE, grown in rich medium with a range of arabinose concentrations. Increasing PDE production corresponds with decreasing intracellular c-di-GMP. (A and B) Measurements of β-galactosidase activity were done with at least three independent biological samples, and the means and standard error are shown. * *P* < 0.05, *** *P* < 0.001, n.s. = not significant by one-way ANOVA and Bonferroni’s multiple comparison test.

The data presented above suggest that c-di-GMP signaling through Vc1 promotes downstream gene expression. We therefore determined the effect of altering the intracellular c-di-GMP concentration on GbpA production, using plasmid-borne, inducible diguanylate cyclase (pDGC) and phosphodiesterase (pPDE) enzymes to artificially increase or decrease intracellular c-di-GMP levels in *V*. *cholerae*, respectively [7,25]. For this study, the *gbpA* 5’UTR containing Vc1 was fused to a *lacZ* reporter gene and placed under the control of the constitutive P_*lacUV5*_ promoter [38]. We chose a constitutive promoter based on findings that c-di-GMP levels can impact the activity of the native *gbpA* promoter [33]. This translational reporter was integrated into the *V*. *cholerae* chromosome within the native *lacZ* gene, generating the “P_*lacUV5*_-Vc1UTR-*lacZ*” strain. Thus, the response of the Vc1 element to c-di-GMP could be assessed in isolation from the native promoter and downstream gene, in single copy. A promoterless *gbpA* 5’ Vc1UTR-lacZ fusion strain served as a control to ensure that sequences within the 5’ UTR do not initiate transcription. pDGC, pPDE, pPDE^mut^ and control vector were introduced into the reporter strains to allow manipulation of c-di-GMP as described [7,25]. As expected, the strains with the promoterless fusion had low β-galactosidase activity, regardless of c-di-GMP level (S3 Fig). The *V*. *cholerae* P_*lacUV5*_-Vc1UTR-*lacZ* strain with reduced intracellular c-di-GMP (pPDE, + L-arabinose) showed a greater than 50% decrease in β-galactosidase activity compared to *V*. *cholerae* with wild-type levels of c-di-GMP (vector, + L-arabinose) (Fig 5A). Conversely, increasing intracellular c-di-GMP through the ectopic production of a diguanylate cyclase (pDGC, VCA0956) significantly increased β-galactosidase activity (S3 Fig). In addition, expression from pPDE^mut^, in which a glutamic acid residue essential for PDE activity of the VieA PDE has been mutated to an alanine (VieA-E170A) [7], resulted in no change in β-galactosidase activity, indicating that altered expression is specifically due to decreased c-di-GMP (S3 Fig).

Next, we examined *V*. *cholerae* strains containing a derivative of P_*lacUV5*_-Vc1UTR-*lacZ* in which the Vc1 sequence contains the G12T mutation (P_*lacUV5*_-Vc1UTR^G12T^-*lacZ*) or A39T mutation (P_*lacUV5*_-Vc1UTR^A39T^-*lacZ*) for altered gene expression in response to c-di-GMP depletion. In the Vc1^G12T^ reporter strain, β-galactosidase activity is reduced compared to that of *V*. *cholerae* with the wild type P_*lacUV5*_-Vc1UTR*-lacZ* fusion even in the absence of c-di-GMP manipulation (Fig 5A, grey bars). When compared to wild type c-di-GMP levels, depleting c-di-GMP does not further reduce reporter activity (Fig 5A). In contrast, the Vc1^A39T^ reporter strain showed activity comparable to the wild type reporter strain in the absence of c-di-GMP manipulation, and activity remained at those levels when c-di-GMP levels were depleted. These results suggest that decreasing c-di-GMP availability in the cell has a similar effect as the G12 mutation, supporting a role for Vc1 in *gbpA* expression in response to c-di-GMP. Furthermore, the A39T mutation did not abrogate downstream gene expression, consistent with the in vivo data shown in Fig 2.

Mutation of a residue important for ligand binding by Vc1 and depletion of the c-di-GMP ligand both resulted in the significant reduction of downstream gene expression. We thus examined the responsiveness of Vc1 to a range of c-di-GMP concentrations. This was done by growing the P_*lacUV5*_-Vc1UTR-*lacZ* reporter strain containing pPDE in medium with different concentrations of arabinose inducer; the consequent range of PDE production is expected to result in a range of c-di-GMP levels. The P_*lacUV5*_-Vc1UTR-*lacZ* reporter strain with vector served as a negative control, and the addition of arabinose did not affect β-galactosidase activity in this strain. For the strain bearing pPDE, β-galactosidase activity decreased in a dose-dependent fashion, with a gradual reduction in activity as PDE production increased and c-di-GMP level decreased (Fig 5B). Thus, the Vc1 element may modulate downstream gene expression in accordance with c-di-GMP levels in the cell.

## Discussion

Cyclic diguanylate (c-di-GMP) has pleiotropic effects on bacterial physiology and broadly impacts gene expression. With the exception of a handful of transcription factors that have been shown to regulate gene expression in response to c-di-GMP, the molecular basis of gene regulation by c-di-GMP is poorly understood. The identification of c-di-GMP specific riboswitches distributed widely among bacterial genomes positions these regulatory RNA domains to serve as important c-di-GMP effectors modulating downstream gene expression. Elegant studies have biochemically characterized a representative GEMM riboswitch, Vc2 from *Vibrio cholerae*, but the functionality of c-di-GMP riboswitches in their native genetic contexts has been largely unexplored. This study investigates the *in vivo* and *in vitro* functionality of the putative c-di-GMP riboswitch Vc1 from *V*. *cholerae*.

Several lines of evidence support a role for Vc1 in sensing c-di-GMP in *V*. *cholerae*. Of key importance is the finding that the Vc1 sequence itself influences gene expression *in cis*, in response to changes in c-di-GMP. Specifically, isolating the Vc1 sequence by fusing it to a reporter gene (*lacZ*), placing it under the transcriptional control of a heterologous, constitutive promoter, and integrating it in the *V*. *cholerae* chromosome rendered reporter activity regulatable by c-di-GMP. Moreover, mutating a single residue in the Vc1 sequence, the G12 nucleotide predicted to be involved in c-di-GMP binding, eliminated regulation of downstream expression by c-di-GMP. These findings are supported by independent experiments showing that the same mutation at the native *gbpA* locus inhibited downstream gene expression. SHAPE and equilibrium dialysis studies using Vc1 RNA corroborate a direct interaction between Vc1 and c-di-GMP. In addition, equilibrium dialysis assays showed that the G12 mutation that reduced downstream gene expression similarly diminished the interaction between Vc1 and c-di-GMP, and mutation of two other conserved putative ligand-binding residues in Vc1, A39 and C104, abolished the ability of Vc1 to interact with c-di-GMP. Together these results support a role for the Vc1 element in regulation of *gbpA* expression in response to c-di-GMP, and point to Vc1 functioning as an “on” switch in response to c-di-GMP.

However, some of our results suggest that Vc1 does not fully behave as a canonical GEMM riboswitch. For example, the equilibrium dialysis data suggest that despite conservation of key residues, the interaction between c-di-GMP and Vc1 is much weaker than that with Vc2. This is supported by assessments of c-di-GMP binding using a differential radial capillary action of ligand assay (DRaCALA), which showed binding of c-di-GMP^32^ by Vc2 RNA as described previously, but failed to show a substantial interaction between Vc1 and c-di-GMP^32^ (data not shown). In addition, previous analyses of Vc2 showed that mutating G20 reduced binding of c-di-GMP by 30,000-fold [37], but mutating the equivalent G12 residue did not fully eliminate c-di-GMP binding according to equilibrium dialysis. The high amount of RNA needed for these assays suggests only a fraction of the RNA assumed a conformation competent for ligand binding under the conditions used. Further differentiating Vc1 from Vc2, the addition of excess, non-radiolabelled competitor c-di-GMP to the equilibrium chambers led to displacement of pre-bound c-di-GMP^32^ from Vc1, but not from Vc2. These results suggest that binding of c-di-GMP by Vc1 is reversible, which may allow for more rapid and dynamic modulation of GbpA production. Together, *in vitro* and *in vivo* analyses of Vc1 suggest that although it may be a poor c-di-GMP binding riboswitch relative to Vc2, the Vc1 sequence is capable of regulating downstream gene expression in response to c-di-GMP. Other putative c-di-GMP riboswitches may also vary in their interactions with and responses to c-di-GMP.

Comparing *in vitro* and *in vivo* data, the Vc1 G12T, A39T and C104G mutations significantly reduce or eliminate the interaction with c-di-GMP *in vitro*; however, downstream gene expression *in vivo* was only significantly decreased by the G12T mutation. One interpretation of these results is that the involvement of particular residues in binding c-di-GMP may not directly correlate to a role in downstream gene expression. Alternatively, it is possible that the G12T mutant mimics the Vc1 “off” state, whereas the A39T and C104G mutants mimic the Vc1 “on” state. Thus, while each mutation would decrease the interaction of Vc1 with c-di-GMP, divergent effects on downstream gene expression could occur. This idea is supported by *in vivo* experiments using isolated wild type and mutant Vc1 sequences fused to a *lacZ* reporter, under the control of a heterologous, constitutive promoter; here, expression under the control of the Vc1^A39T^ UTR was comparable to that of the wild type riboswitch and remained so upon perturbation of c-di-GMP levels. These results are consistent with the finding that the A39T mutation on the *V*. *cholerae* chromosome did not attenuate GbpA levels. The Vc2 riboswitch, while well studied *in vitro*, has not been examined for functionality *in vivo*, so it is unclear what effect the G20, A47 and C92 mutations have on downstream gene expression for comparison.

*In vivo* experiments using the PlacUV5-Vc1UTR-*lacZ* fusion, which isolates the regulatory function of Vc1, revealed that Vc1 is capable of controlling gene expression in a dose-dependent manner according to a spectrum of c-di-GMP levels in the cell. The ability of Vc1 to respond to a range of c-di-GMP levels is consistent with prior work using a reporter controlled by an engineered c-di-GMP riboswitch, which also suggested that reporter activity varied depending on intracellular c-di-GMP levels [39]. In *Salmonella* Typhimurium, various proteins that sense c-di-GMP to control motility and biofilm production also bind c-di-GMP with different affinities [40]. This study suggests that sequential activation of these receptors to changing c-di-GMP concentrations is important to mediate a progressive response. *V*. *cholerae* encodes 61 genes with predicted functions as PDE and/or DGC enzymes, a subset of which likely modulate c-di-GMP levels to presumably achieve a range of c-di-GMP levels in response to environmental stimuli. Therefore, the ability of Vc1 to respond to varying intracellular c-di-GMP concentrations may allow for rapid changes in *gbpA* expression according to extracellular cues.

Regulation via a riboswitch typically occurs as a result of ligand-induced conformational changes in the RNA structure [41,42]. Most commonly, riboswitches regulate gene expression through the formation of structures that control either premature transcriptional termination or translational initiation. There are no apparent Rho-independent transcription terminator or anti-Shine Delgarno sequences between Vc1 and the *gbpA* open reading frame, so the mechanism by which Vc1 regulates *gbpA* expression is unclear. Future work will determine whether Vc1 controls transcriptional read-through, translation initiation or transcript stability. The mechanism by which the Vc2 riboswitch regulates VC1722 expression is similarly unknown.

Several lines of evidence suggest that c-di-GMP levels change in *V*. *cholerae* during transitions between its native aquatic environment and the host intestine. For example, *V*. *cholerae* forms biofilms on surfaces in the environment, such as the chitin, and numerous studies have shown that biofilm formation is positively regulated by c-di-GMP [25,43,44]. Furthermore, increased intracellular c-di-GMP enhances the binding of *V*. *cholerae* to chitin *in vitro* in a process dependent on the hemagglutinin FrhA [45]. Conversely, reduction of c-di-GMP is required to promote bacterial motility and increase expression of virulence factors in the intestinal tract [25,43,44]. In the context of *V*. *cholerae* biology, the effect of c-di-GMP sensing by Vc1 to augment GbpA production may contribute to colonization of environmental (chitin) and host surfaces. Yet determining the role of c-di-GMP in controlling c-di-GMP—regulated processes of *V*. *cholerae* is complicated by the finding that the *gbpA* promoter is negatively regulated by c-di-GMP [33]. Additional work is needed to define the roles of Vc1 and Vc2 in modulating c-di-GMP-regulated processes in response to extracellular cues in *V*. *cholerae*.

## Supporting Information

S1 Fig The translational start site of *gbpA*. (DOC)

S2 Fig GTP does not induce structural changes in Vc1 RNA. (DOC)

S3 Fig The effect of PDE and DGC gene expression on reporter activity is specifically due to altered c-di-GMP. (DOC)

S1 File Supplemental Methods. (DOC)

S1 Table Strains and Plasmids used in this study. (DOC)

S2 Table Primers used in this study. (DOC)
