# Supplementary material for: The RNA Domain Vc1 Regulates Downstream Gene Expression in Response to Cyclic Diguanylate in Vibrio cholerae
Source: PLoS One. 2016 Feb 5;11(2):e0148478. doi: 10.1371/journal.pone.0148478 (PMC4744006; doi:10.1371/journal.pone.0148478)
Supplement: S1 Fig — (DOC) [file pone.0148478.s001.doc]

**
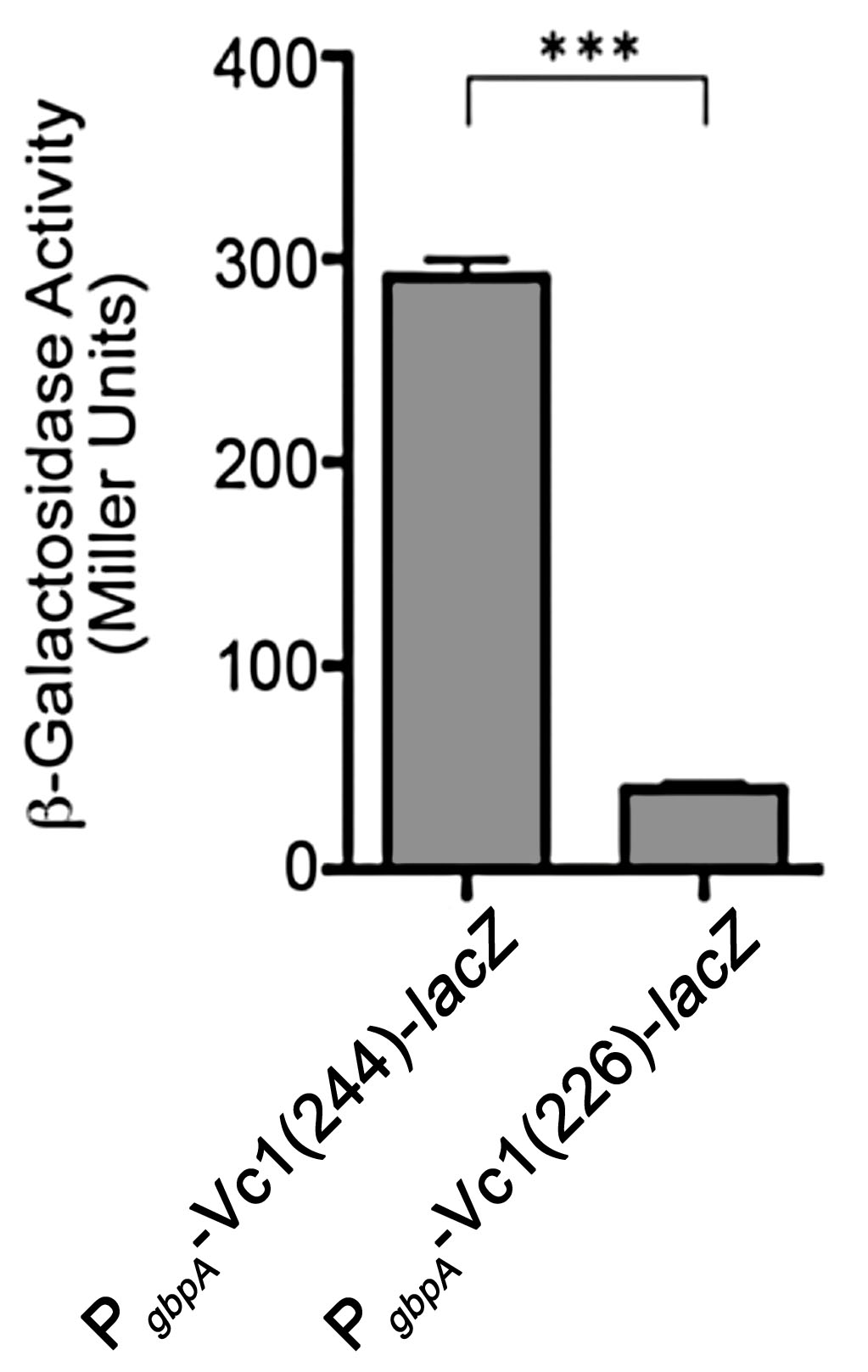
**

**Figure S1.**  The translation start site of *gbpA* is at position +244. To create a translational fusion reporter plasmid, we first identified the methionine start codon in the *gbpA* ORF. The *E. coli lacZ* gene was substituted for the *gbpA* ORF beginning at either the methionine codon at position +226 (the annotated translational start site), or the methionine codon at position +244, in each case using the native translational initiation sequence. The β-galactosidase activity of *V. cholerae* strains with *lacZ* translational fusions to P*_gbpA_*-Vc1(226) or P*_gbpA_*-Vc1(244) that encompass the *gbpA* promoter and the 225 bases or 243 bases of the 5’ UTR, respectively, was measured using a Miller assay. β-galactosidase activity was approximately 7-fold higher when the second methionine was included in the fusion, suggesting that the annotated translational start site is incorrect and the 5’UTR of *gbpA* is 243 bp long. *** *P* < 0.001 by unpaired t-test.
