# Supplementary material for: The RNA Domain Vc1 Regulates Downstream Gene Expression in Response to Cyclic Diguanylate in Vibrio cholerae
Source: PLoS One. 2016 Feb 5;11(2):e0148478. doi: 10.1371/journal.pone.0148478 (PMC4744006; doi:10.1371/journal.pone.0148478)
Supplement: S2 Fig — (DOC) [file pone.0148478.s002.doc]

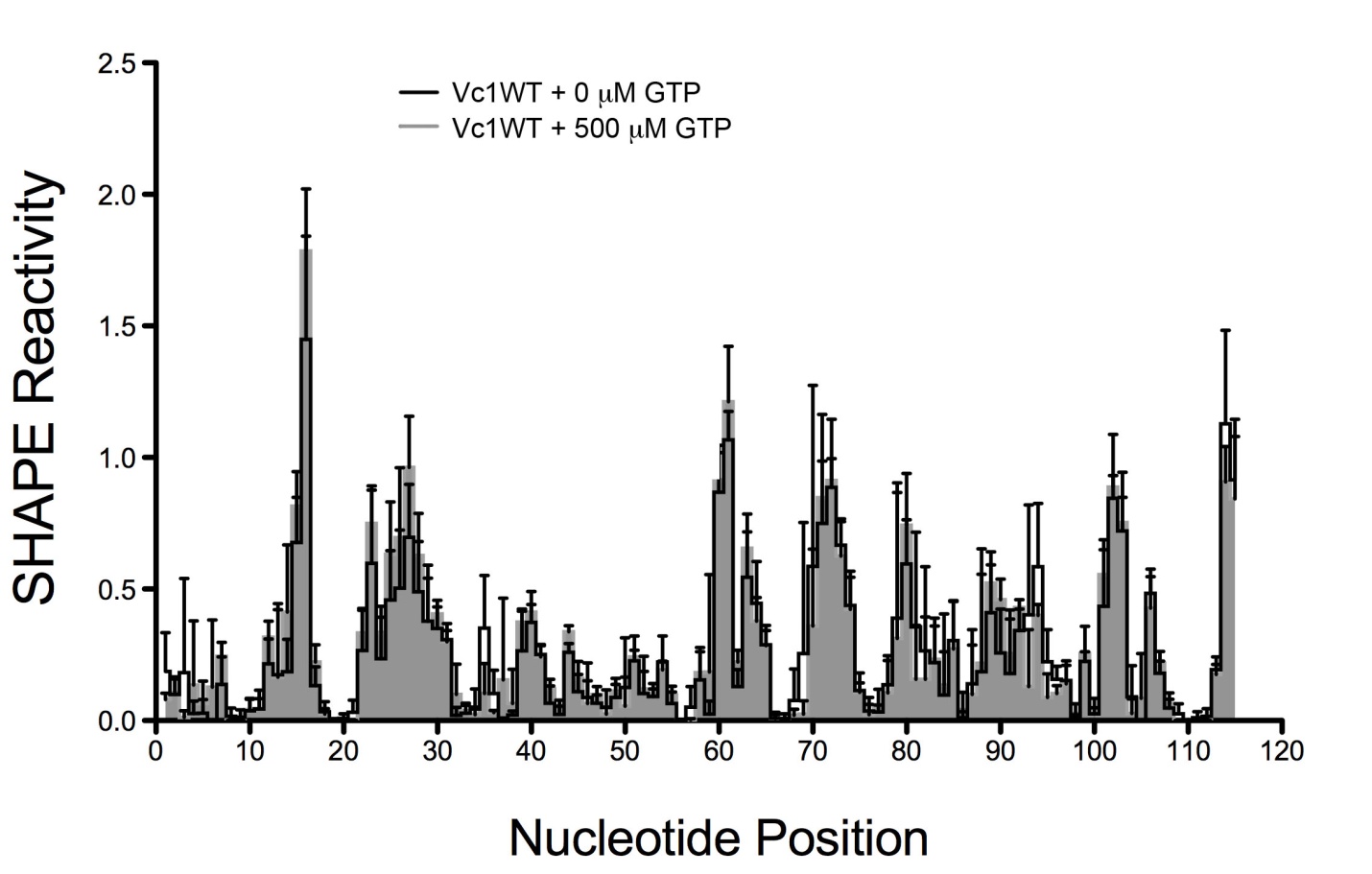


**Figure S2.** GTP does not induce structural changes in Vc1 RNA. *In vitro* transcribed Vc1 RNA was incubated with or without 500 µM GTP and analyzed by SHAPE. SHAPE reactivity values were obtained by averaging three independent experiments. The data were analyzed by unpaired t-test comparing SHAPE reactivity values for each nucleotide position; no significant differences were identified.
