# Supplementary material for: The RNA Domain Vc1 Regulates Downstream Gene Expression in Response to Cyclic Diguanylate in Vibrio cholerae
Source: PLoS One. 2016 Feb 5;11(2):e0148478. doi: 10.1371/journal.pone.0148478 (PMC4744006; doi:10.1371/journal.pone.0148478)
Supplement: S3 Fig — (DOC) [file pone.0148478.s003.doc]

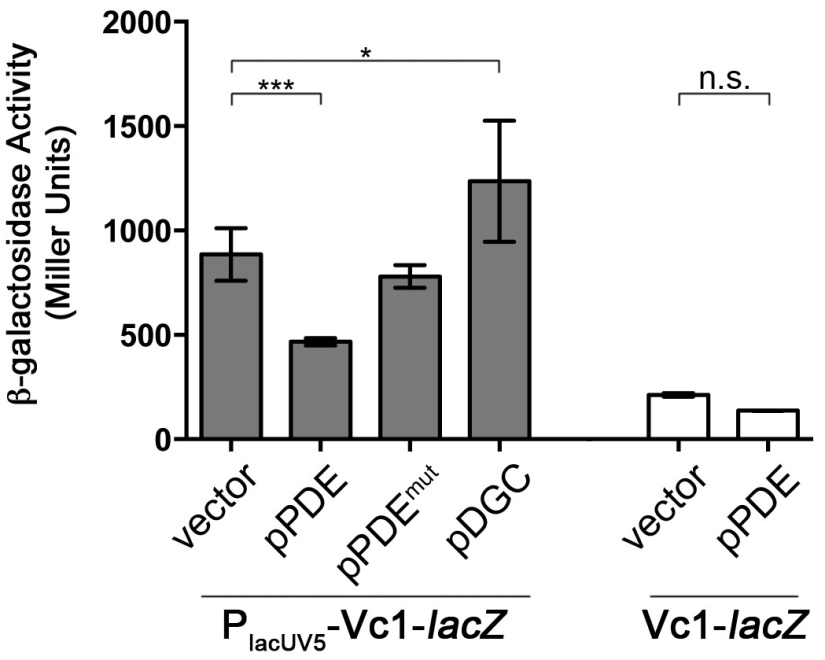


**Figure S3.** The effect of PDE (VieA) and DGC (VCA0956) gene expression on P*_lacUV5_*-UTR-*lacZ* reporter activity is specifically due to altered c-di-GMP. The β-galactosidase activity was measured for *V. cholerae* strains with chromosomal translational fusions of *E. coli lacZ* to the wild type *gbpA* 5’ UTR, with either vector (wild-type c-di-GMP level), pPDE (reduced c-di-GMP), pPDE^mut^ (inactive PDE, c-di-GMP) or pDGC (DGC VCA0956, increased c-di-GMP). Transcription initiation was controlled by the constitutive P*_lacUV5_* promoter. The β-galactosidase activity of control strains containing a promoterless UTR-*lacZ* fusion inserted in the chromosome, with either vector (wild type c-di-GMP) or pPDE (reduced c-di-GMP) confirm that no element in the UTR drive transcription. All strains were grown in rich medium with 0.2% L-arabinose to induce gene expression. Three independent experiments were done, and the means and standard deviations are shown. * *P* < 0.05, *** *P* < 0.001 by two-way ANOVA and Tukey’s multiple comparisons test.
