# Supplementary material for: The RNA Domain Vc1 Regulates Downstream Gene Expression in Response to Cyclic Diguanylate in Vibrio cholerae
Source: PLoS One. 2016 Feb 5;11(2):e0148478. doi: 10.1371/journal.pone.0148478 (PMC4744006; doi:10.1371/journal.pone.0148478)
Supplement: S1 File — (DOC) [file pone.0148478.s004.doc]

**Supplemental Methods**

*Genetic manipulations and strain construction*. To make deletions by allelic exchange, using genomic DNA from *V. cholerae* C6706, ~800 bp fragments upstream and downstream of the sequences to be deleted were amplified by PCR using primers named according to the pattern geneF1 + geneR1 for the upstream region of homology and geneF2 + geneR2 for the downstream region of homology. The primers introduced restriction sites (underlined sequences in Table S2) that allowed ligation of the two PCR products to each other and into the suicide vector pCVD442. The ligations were transformed into DH5αλpir by electroporation and transformed colonies were identified on LB-Amp agar. The desired clones that contain the upstream and downstream fragments were identified by PCR using primers geneF1 + geneR2 and/or pCVDseqF + pCVDseqR, which flank the multiple cloning site of pCVD442. For deletion of *gbpA*, the resulting pCVD442::Δ*gbpA* plasmid was transformed into Sm10λpir by electroporation and grown on LB-Amp agar. The resulting strain was mated with *V. cholerae* C6706 on LB agar for ~8hrs, then transconjugants were selected on LB-Sm-Amp agar. Sm-Amp-resistant transconjugants were grown in LB broth in the absence of selection for 8 hours, then dilutions of the cultures were plated on LB-Sm agar containing 10% sucrose. Sucrose-resistant, Amp-sensitive colonies were screened for the relevant deletion by PCR using the corresponding geneF0 + geneR2 primers. To create a *V. cholerae* strain with a deletion of Vc1 (∆Vc1), fragments upstream and downstream to Vc1 were amplified using Vc1F + placP2R and placP2F + Vc1R primers, respectively, using genomic DNA from *V. cholerae* C6706 as the template. The upstream and downstream fragments were digested with the relevant enzymes and ligated into pCVD442. The ligation was transformed into DH5αλpir by electroporation and transformed colonies were identified on LB-Amp agar. The desired clone was identified by PCR using primers pCVDseqF + pCVDseqR. The resulting pCVD442::ΔVc1 plasmid was transformed into Sm10λpir by electroporation, then mated with *V. cholerae* C6706 on LB agar. Mutants containing the Vc1 deletion were made and identified as described above.

Point mutations in Vc1 and Vc2 were generated by incorporating the desired nucleotide changes into self-complementary primers. These primers were used in separate PCRs to amplify two, overlapping fragments which were then cloned into the allelic exchange vector pCVD442. Genomic DNA from *V. cholerae* C6706 was used in all cases. Specifically, to generate the “P1” mutation consisting of CACAC to GTGTG mutations in the putative P1 stem of Vc1, the upstream fragment was amplified using Vc1F + VC1BR and the downstream fragment with primers Vc1BF + Vc1R. A similar technique was used to create point mutations in Vc1. For the Vc1^G12T^ mutant, primer combinations Vc1F + Vc1gTR and Vc1gTF + Vc1R were used to amplify the two overlapping fragments; for the Vc1^A39T^ mutant, Vc1F + Vc1aTR and Vc1aTF + Vc1R were used; for the Vc1^C104G^ mutant, Vc1F + Vc1cGR and Vc1cGF + Vc1R were used; and for Vc2^G20T^, Vc2G20TF1 + Vc2G20TR1 and Vc2G20TF2 + Vc2G20TR2 were used. For all four Vc1 mutants, the resulting pairs of fragments were combined and used as the template in PCR reactions with Vc1F and Vc1R as primers, to yield a spliced product containing the desired nucleotide changes. For the Vc2 mutation, Vc2G20TF1 + Vc2G20TR2 were used to obtain the spliced product. The spliced PCR products were digested with the appropriate restriction enzymes, for which restriction sites were introduced with the outer primers, then cloned into similarly digested pCVD442. Plasmids containing insert were identified by PCR, and point mutations were confirmed by sequencing. The confirmed plasmids were introduced into *V. cholerae* C6706 via SM10λPir, and the desired *V. cholerae* mutants were identified, as described above. All mutations to the *V. cholerae* chromosome were confirmed by sequencing.

The plasmid-borne *lacZ* reporter constructs were made by amplifying the 5’ UTR of *gbpA* from genomic DNA of *V. cholerae* C6706 “Vc1^WT^”, Vc1^P1^, Vc1^G12T^, Vc1^A39T^, or Vc1^C104G^ using primers gbpArbF and gbpAPrR and digested with *Eco*RI and *Sal*I. The Vc1 fragments were ligated into similarly-digested pP*lac*thiM#2-*lacZ*, then ligations were transformed into DH5α. Amp-resistant clones were screened by PCR using primers pLacSeq + gbpAPrR. The resulting plasmids have Vc1 (wild type or mutant) as a translational fusion to *lacZ*, with the *lac* promoter driving transcription. Likewise, the pP*gbpA*-Vc1-*lacZ* and pP*gbpA*-∆Vc1-*lacZ* plasmids were constructed by amplifying P*gbpA*-∆Vc1-Vc1 from *V. cholerae* C6706 and ΔVc1, respectively, by PCR using gbpAP2F + gbpAR2. The PCR products were digested with *Eco*RI and *Sal*I and ligated into pP*lac*thiM#2-*lacZ* digested with the same enzymes. The ligation reaction was transformed into DH5α cells by electroporation, and resulting Amp-resistant colonies were screened with primers pLacSeq + gbpAR2.

To make the chromosomal Vc1 reporter strains, the pP*lac*-Vc1-*lacZ* and pP*lac*-Vc1^G12T^-*lacZ* vector backbones were amplified to exclude the P*lac* promoter gbpArbF + PlacZR3, which introduced *Eco*RI and *Sph*I sites, respectively. The PlacUV5 promoter was amplified from p2UY35A using PlacUV5F + PlacUV5R, which also introduced *Sph*I and *Eco*RI sites, respectively [1]. The PlacUV5 promoter and Vc1-*lacZ* vector (now lacking Plac) were digested with *Sph*I and *Eco*RI, ligated together, and cloned in *E. coli* DH5α, yielding plasmid pPlacUV5-Vc1UTR-*lacZ* and pPlacUV5-Vc1UTR^G12T^-*lacZ*.

The resulting plasmids, pPlacUV5-UTR-*lacZ* and pPlacUV5-UTR^Vc1G12T^-*lacZ,* were used as the template for amplification of the fusions with primers PlacF5 + PlacR5, which introduced *Stu*I and *Not*I sites, respectively. The PCR products were digested with *Stu*I and *Not*I and cloned into similarly digested pJL1, a suicide vector which allows targeting of DNA into the *lacZ* gene of *V. cholerae* [2]. The pJL1:: PlacUV5-UTR-*lacZ* plasmid was used as template for creation of the Vc1A39T derivative, in which the PlacUV5-UTR^Vc1A39T^ was generated by splicing by overlap extension using primers PlacF5 + Vc1aTR for the upstream fragment and Vc1aTF + PlacR5 for the downstream fragment. The spliced product was cloned into pJL1 via the *Stu*I and *Not*I sites. The pJL1 constructs were introduced into *V. cholerae* C6706 by conjugation via SM10λpir *E. coli*. Incorporation of the fusions in the *V. cholerae lacZ* gene was achieved by standard allelic exchange methods and confirmed by PCR with lacZsF +lacZsR and by sequencing. The resulting chromosomal reporter strains were transformed with vector, pPDE and pPDE^mut^ plasmids by electroporation to allow manipulation of c-di-GMP [3,4].

References:

1. Marden JN, Diaz MR, Walton WG, Gode CJ, Betts L, Urbanowski ML, et al. An unusual CsrA family member operates in series with RsmA to amplify posttranscriptional responses in *Pseudomonas aeruginosa*. Proc Natl Acad Sci U S A. 2013;110: 15055-15060. doi: 10.1073/pnas.1307217110 [doi].

2. Butterton JR, Ryan ET, Acheson DW, Calderwood SB. Coexpression of the B subunit of Shiga toxin 1 and EaeA from enterohemorrhagic *Escherichia coli* in *Vibrio cholerae* vaccine strains. Infect Immun. 1997;65: 2127-2135.

3. Tischler AD, Camilli A. Cyclic diguanylate (c-di-GMP) regulates *Vibrio cholerae* biofilm formation. Mol Microbiol. 2004;53: 857-69.

4. Tamayo R, Tischler AD, Camilli A. The EAL domain protein VieA is a cyclic diguanylate phosphodiesterase. J Biol Chem. 2005;280: 33324-30.
