# Supplementary material for: The RNA Domain Vc1 Regulates Downstream Gene Expression in Response to Cyclic Diguanylate in Vibrio cholerae
Source: PLoS One. 2016 Feb 5;11(2):e0148478. doi: 10.1371/journal.pone.0148478 (PMC4744006; doi:10.1371/journal.pone.0148478)
Supplement: S1 Table — (DOC) [file pone.0148478.s005.doc]

Table S1. Strains and plasmids used in this study.

| **Strain** | **Strain Description** | | **Reference** |
| --- | --- | --- | --- |
| *Escherichia coli* |  | |  |
| DH5α | F^–^ φ80*lacZ* ΔM15 Δ(*lacZYA-argF*)*U169* *recA1 endA1 hsdR17* (rK-, mK+) *phoA supE*44 *thi-1 gvrA96 relA1 λ- tonA* | | Invitrogen;  [1] |
| DH5αλpir | F^–^ Δ(*lacZYA-argF*)*U169* *recA1 endA1 hsdR17 supE44 thi-1 gvrA96 relA1 λpir* | | [1] |
| SM10λpir | *thi thr leu tonA lacY supE recA*::RPA-2-Te::Mu *λpir*R6K, Km^r^ | | [2] |
|  |  | |  |
| *Vibrio cholerae* |  | |  |
|  | C6706 (O1 El Tor) | | [3] |
|  | C6706 pBAD33 | | This study |
|  | C6706 pBAD33::*vieA* (pPDE) | | This study |
|  | C6706 pBAD33::*vieA*E170A (pPDE^mut^) | | This study |
|  | C6706 ∆*lacZ* | | [4] |
|  | C6706 ∆*lacZ* pP*lac*-Vc1-*lacZ* | | This study |
|  | C6706 ∆*lacZ* pP*lac*-Vc1^P1^-*lacZ* | | This study |
|  | C6706 ∆*lacZ* pP*lac*-Vc1^A39T^-*lacZ* | | This study |
|  | C6706 ∆*lacZ* pP*lac*-Vc1^G12T^ -*lacZ* | | This study |
|  | C6706 ∆*lacZ* pP*lac*-Vc1^C104G^-*lacZ* | | This study |
|  | C6706 ∆*gbpA* (VCA0811) | | This study |
|  | C6706 Vc1^P1^ | | This study |
|  | C6706 Vc1^G12T^ | | This study |
|  | C6706 Vc1^A39T^ | | This study |
|  | C6706 Vc1^C104G^ | | This study |
|  | C6706 Vc1^G12T^ pBAD33 | | This study |
|  | C6706 Vc1^G12T^ pBAD33::*vieA* (pPDE) | | This study |
|  | C6706 PlacUV5-Vc1UTR-*lacZ* | | This study |
|  | C6706 PlacUV5-Vc1UTR-*lacZ* pBAD33 | | This study |
|  | C6706 PlacUV5-Vc1UTR-*lacZ* pBAD33::*vieA* (pPDE) | | This study |
|  | C6706 PlacUV5-Vc1UTR-*lacZ* pBAD33::*vieA*E170A (pPDE^mut^) | | This study |
|  | C6706 PlacUV5-Vc1UTR^Vc1G12T^-*lacZ* | | This study |
|  | C6706 PlacUV5-Vc1UTR^Vc1G12T^-*lacZ* pBAD33 | | This study |
|  | C6706 PlacUV5-Vc1UTR^Vc1G12T^-*lacZ* pBAD33::*vieA* (pPDE) | | This study |
|  | C6706 PlacUV5-Vc1UTR^Vc1G12T^-*lacZ* pBAD33::*vieA*E170A (pPDE^mut^) | | This study |
|  | C6706 PlacUV5-Vc1UTR^Vc1A39T^-*lacZ* pBAD33::*vieA* (pPDE) | | This study |
|  |  | |  |
| *Plasmids* |  |  |  |
| pP*lac*thiM#2-*lacZ* | The TPP riboswitch encoded in the 5‘ UTR of *E. coli thiM* was mutated to function as an ON switch and cloned into pP*lac*-*lacZ*. Origin of replication *colEI.* Amp^R^ | | [5] |
| pP*lac*-Vc1-*lacZ* | Vc1 allele from C6706 cloned into  plasmid pP*lac*thiM#2-*lacZ* | | This study |
| pP*lac*-Vc1^P1^-*lacZ* | Vc1^P1^ allele cloned into plasmid pP*lac*thiM#2-*lacZ* | | This study |
| pP*lac*-Vc1^A39T^*-lacZ* | Vc1^A39T^ allele cloned into plasmid pP*lac*-Vc1-*lacZ* | | This study |
| pP*lac*-Vc1^G12T^-*lacZ* | Vc1^G12T^ allele cloned into plasmid pP*lac*-Vc1-*lacZ* | | This study |
| pP*lac*-Vc1 ^C104G^-*lacZ* | Vc1^C104G^ allele cloned into plasmid pP*lac*-Vc1-*lacZ* | | This study |
| pBAD33 | Expression vector, P*_ara_* promoter, Cm^R^ | | [6] |
| pBAD33::*vieA* | *vieA* in pBAD33 (“pPDE”) | | [7] |
| pBAD33::*vieA*E170A | *vieA*E170A (allele encoding enzymatically inactive VieA) (“pPDE^mut^”) | | [7] |
| pCVD442 | *oriR6K* plasmid with a polylinker, *mobRP4*, *bla*, and *sacB* | | [8] |
| pCVD442::Δ*gbpA* | Allelic exchange vector for in-frame deletion of *gbpA* | | This study |
| pCVD442::Vc1^P1^ | Allelic exchange vector for mutation of P1 stem | | This study |
| pCVD442::Vc1^G12T^ | Allelic exchange vector for mutation of nucleotide G12 in Vc1 | | This study |
| pCVD442::Vc1^A39T^ | Allelic exchange vector for mutation of nucleotide A39 in Vc1 | | This study |
| pCVD442::Vc1^C104G^ | Allelic exchange vector for mutation of nucleotide C104 in Vc1 | | This study |
| pCVD442::Vc2^G20T^ | Allelic exchange vector for mutation of nucleotide G20 in Vc2 | | This study |
| pJL1 | Allelic exchange vector for integration into *V. cholerae lacZ* gene | | [9] |
| p2UY35A | Template used for amplification of PlacUV5 promoter | | [10] |
| pJL1:: PlacUV5-Vc1UTR-*lacZ* | Allelic exchange vector for integration of PlacUV5-Vc1UTR-*lacZ* translational fusion into *V. cholerae lacZ* gene (WT 5’ UTR of *gbpA* containing Vc1) | | This study |
| pJL1:: PlacUV5-Vc1UTR^G12T^-*lacZ* | Allelic exchange vector for integration of PlacUV5-Vc1UTR^G12T^-*lacZ* translational fusion into *V. cholerae lacZ* gene (mutant 5’ UTR of *gbpA* containing Vc1) | | This study |
| pJL1:: PlacUV5-Vc1UTR^A39T^-*lacZ* | Allelic exchange vector for integration of PlacUV5-Vc1UTR^A39T^-*lacZ* translational fusion into *V. cholerae lacZ* gene (mutant 5’ UTR of *gbpA* containing Vc1) | | This study |

**References:**

1. Hanahan D. Studies on transformation of *Escherichia coli* with plasmids. J Mol Biol. 1983;166: 557-80.

2. Miller VL, Mekalanos JJ. A novel suicide vector and its use in construction of insertion mutations: osmoregulation of outer membrane proteins and virulence determinants in *Vibrio cholerae* requires *toxR*. J Bacteriol. 1988;170: 2575-83.

3. Thelin KH, Taylor RK. Toxin-coregulated pilus, but not mannose-sensitive hemagglutinin, is required for colonization by *Vibrio cholerae* O1 El Tor biotype and O139 strains. Infect Immun. 1996;64: 2853-6.

4. Tamayo R, Patimalla B, Camilli A. Growth in a biofilm induces a hyperinfectious phenotype in *Vibrio cholerae*. Infect Immun. 2010;78: 3560-3569. doi: 10.1128/IAI.00048-10.

5. Nomura Y, Yokobayashi Y. Reengineering a natural riboswitch by dual genetic selection. J Am Chem Soc. 2007;129: 13814-13815. doi: 10.1021/ja076298b [doi].

6. Guzman LM, Belin D, Carson MJ, Beckwith J. Tight regulation, modulation, and high-level expression by vectors containing the arabinose PBAD promoter. J Bacteriol. 1995;177: 4121-4130.

7. Tischler AD, Camilli A. Cyclic diguanylate (c-di-GMP) regulates *Vibrio cholerae* biofilm formation. Mol Microbiol. 2004;53: 857-69.

8. Donnenberg MS, Kaper JB. Construction of an *eae* deletion mutant of enteropathogenic *Escherichia coli* by using a positive-selection suicide vector. Infect Immun. 1991;59: 4310-7.

9. Butterton JR, Ryan ET, Acheson DW, Calderwood SB. Coexpression of the B subunit of Shiga toxin 1 and EaeA from enterohemorrhagic *Escherichia coli* in *Vibrio cholerae* vaccine strains. Infect Immun. 1997;65: 2127-2135.

10. Marden JN, Diaz MR, Walton WG, Gode CJ, Betts L, Urbanowski ML, et al. An unusual CsrA family member operates in series with RsmA to amplify posttranscriptional responses in *Pseudomonas aeruginosa*. Proc Natl Acad Sci U S A. 2013;110: 15055-15060. doi: 10.1073/pnas.1307217110 [doi].
