# Supplementary material for: The RNA Domain Vc1 Regulates Downstream Gene Expression in Response to Cyclic Diguanylate in Vibrio cholerae
Source: PLoS One. 2016 Feb 5;11(2):e0148478. doi: 10.1371/journal.pone.0148478 (PMC4744006; doi:10.1371/journal.pone.0148478)
Supplement: S2 Table — (DOC) [file pone.0148478.s006.doc]

Table S2. Primers used in this study.

| **Primer Name** | **Oligonucleotide sequence (5’ to 3’) *** | **Reference** |
| --- | --- | --- |
| gbpAF1 | TTGCATGCTACTCGTCAGGTCTTTGG | This study |
| gbpAR1 | TTGGTACCCATCACAGACTCTTCTTTG | This study |
| gbpAF2 | TTGGTACCTAAGTTATCCTCCCTCTTAC | This study |
| gbpAR2 | TTGAGCTCTTTCTCTGGATGGGAGTC | This study |
| gbpAF0 | GCAAACGGTAGCAAGAAG | This study |
| pCVDseqF | CTGTTGCATGGGCATAAAGTTGCC | This study |
| pCVDseqR | ACACAGGAACACTTAACGGCTGAC | This study |
| gbpAPrR | CGATGTCGACCATCACAGACTCTTCTTTGTTAGC | This study |
| gbpAP2F | CCGAATTCCCGAGTAAAGCATCAACCTTTCATATTG | This study |
| gbpArbF | CCGAATTCAGTAAATTTGCTCTCGGTCACAC | This study |
| Race1a | GACTCGAGTCGACATCGATTTTTTTTTTTTTTTTTA | This study |
| Race1g | GACTCGAGTCGACATCGATTTTTTTTTTTTTTTTTG | This study |
| Race1c | GACTCGAGTCGACATCGATTTTTTTTTTTTTTTTTC | This study |
| Race2 | GACTCGAGTCGACATCG | This study |
| gbpAsp1 | GTGAATCTGAACTTCAGCACC | This study |
| gbpAsp2 | GCACCTTCAGCAACGCATAAG | This study |
| gbpAsp3 | TTGCCAAAGTGTGACCGAGAG | This study |
| gbpAsp4 | ACCAAGAGAGAAAGCGAAGTC | This study |
| placP2R | TTGCATGCTTGCCAAAGTGTGACCGAGAG | This study |
| placP2F | TTGCATGCCAGCTAACAAAGAAGAGTCTGTG | This study |
| Vc1F1 | CCTCTAGATACTCGTCAGGTCTTTGG | This study |
| Vc1R2 | AAGAGCTCCGAGTGTACACGGTATCG | This study |
| Vc1R3 | AGCAACGCATAAGACAAAACG | This study |
| Vc1gTF | CACACTTT**T**GCAAACCCTTTGAAAAAATGG | This study |
| Vc1gTR | AGGGTTTGC**A**AAAGTGTGACCGAGAG | This study |
| Vc1aTF | AATGGGACGC**T**AAGCTTCCGGTCTG | This study |
| Vc1aTR | CGGAAGC**A**TTGCGTCCCATTTTTTC | This study |
| Vc1cGF | GGTTA**G**CAATTCGGTTTATACCG | This study |
| Vc1cGR | ATTG**C**TAACCCCGCTATCC | This study |
| Vc2G20TF1 | CCGAGCTCTCAGAGATGCCTTAATAGCTC | This study |
| Vc2G20TR1 | CAG**T**GCAAACCATTCGAAAG | This study |
| Vc2G20TF2 | GTTTGC**A**CTGTGCGTGA | This study |
| Vc2G20TR2 | GGCCCGGGGCGGCGGATAAAATCATTGC | This study |
| T7linkF | TAATACGACTCACTATAGGGCCTTCGGGCCAACGGTCACACTTTGGCAAACCC | This study |
| T7R | CATCACGCGACAATGGCTGGT | This study |
| gbpAR2 | CGATGTCGACTTTAGGTTGTTTTTTCATCACAG | This study |
| T7Vc1F | TAATACGACTCACTATAGGGAGAAGTAAATTTGCTCTCGGTCACAC | This study |
| T7Vc2F | TAATACGACTCACTATAGGGAGAGGAAAAATGTCACGCACAGG | This study |
| T7Vc2R | CATGCTGTTAGTCTCGGAGTATTG | This study |
| PlacZR3 | GCGCATGCTCACATTAATTGCGTTGCGCTCTCG | This study |
| PlacUV5F | GCGCATGCAAACCCTATGCTAC | This study |
| PlacUV5R | GCGAATTCACACACTATACGAGCCG | This study |
| PlacF5 | CCAGGCCTAATAGGCGTATCACGAGG | This study |
| PlacR5 | CCGCGGCCGCGTCTTTCGACTGAGCCTTTC | This study |
| PlacZsF | AGCACACTAACTACCAGC | This study |
| PlacZsR | GTTTGTATGTGGTGGATGAG | This study |
| RPB2F | CTGTCTCAAGCCGGTTACAA | [1] |
| RPB2R | TTTCTACCAGTGCAGAGATGC | [1] |
| gbpAqF2 | CTCTGGTATCAGTGGATTAGCG | This study |
| gbpAqR2 | GTATTGAATCGCGCCACAGT | This study |
| Vc1BF | GTAAATTTGCTCTCG**GTGTG**TGTTTGGCAAACCCTTTG | This study |
| Vc1BR | CAAAGGGTTTGCCAAACA**CACAC**CGAGAGCAAATTTAC | This study |
| pLacSeq | ATTGTCTCATGAGCGGATAC | This study |
| placZF | ATGCAA**GTCGAC**CTGCTGGATC | This study |

* The underlined sequences represent restriction enzyme recognition sites, and the bold and underline sequences represent point mutations. Italicized sequences encode a 6-histidine tag.

1. Quinones M, Kimsey HH, Waldor MK. LexA cleavage is required for CTX prophage induction. Mol Cell. 2005;17: 291-300.
